# Phenotypic and Genetic Divergence among Poison Frog Populations in a Mimetic Radiation

**DOI:** 10.1371/journal.pone.0055443

**Published:** 2013-02-06

**Authors:** Evan Twomey, Justin Yeager, Jason Lee Brown, Victor Morales, Molly Cummings, Kyle Summers

**Affiliations:** 1 Department of Biology, East Carolina University, Greenville, North Carolina, United States of America; 2 Department of Ecology and Evolutionary Biology, Tulane University, New Orleans, Louisiana, United States of America; 3 Department of Biology, Duke University, Durham, North Carolina, United States of America; 4 La Facultad de Ciencias Biológicas, Universidad de Ricardo Palma, Lima, Peru; 5 Department of Integrative Biology, University of Texas, Austin, Texas, United States of America; Virginia Tech Virginia, United States of America

## Abstract

The evolution of Müllerian mimicry is, paradoxically, associated with high levels of diversity in color and pattern. In a mimetic radiation, different populations of a species evolve to resemble different models, which can lead to speciation. Yet there are circumstances under which initial selection for divergence under mimicry may be reversed. Here we provide evidence for the evolution of extensive phenotypic divergence in a mimetic radiation in *Ranitomeya imitator*, the mimic poison frog, in Peru. Analyses of color hue (spectral reflectance) and pattern reveal substantial divergence between morphs. However, we also report that there is a “transition-zone” with mixed phenotypes. Analyses of genetic structure using microsatellite variation reveals some differentiation between populations, but this does not strictly correspond to color pattern divergence. Analyses of gene flow between populations suggest that, while historical levels of gene flow were low, recent levels are high in some cases, including substantial gene flow between some color pattern morphs. We discuss possible explanations for these observations.

## Introduction

Mimicry between unrelated organisms provides an exceptional example of evolution by natural selection [1]. Müllerian mimicry occurs when two or more toxic species resemble each other, thus sharing the cost of predator learning, which reduces per-capita mortality [2], [3]. In some cases of Müllerian mimicry, a single species has radiated to mimic distinct model species in different geographical areas, resulting in a “mimetic radiation” [4], [5]. For example, in central Peru, the butterflies *Heliconius melpomene* and *H. erato* are co-mimics with two distinct forms: north of the Cordillera Escalera mountains the two species have converged on a ‘rayed’ morph, and south of these mountains the species have converged on the ‘Postman’ morph. Field and laboratory experiments have demonstrated that selection in the context of predation maintains divergence in this system [6].

Previous research indicates that phenotypically distinct populations of the mimic poison frog, *Ranitomeya imitator*, represent a Müllerian mimetic radiation. In each of three different regions, the local populations bear a strong resemblance to a co-occurring, congeneric, toxic model species [5], and selection appears to be acting divergently in this system [7], [8]. Recent evidence indicates that divergence between some *R. imitator* morphs may have occurred concurrently with or prior to that between two putative model morphs in one region [9], but broad phylogenetic analyses [5], [10] clearly indicate that the basal divergence between *R. imitator* morphs was preceded by divergence among distinct model species, and that all the *R. imitator* color pattern morphs are recently diverged members of a southern clade of poison frogs. This supports the hypothesis that *R. imitator* has undergone unilateral ‘advergence’ (see [11] for a discussion of advergence vs. convergence) to resemble distinct models in different geographic regions, and thus in a historical sense can be thought of as the mimic rather than the model, even though the distinction between model and mimic is less clear in Müllerian mimicry than Batesian mimicry.

This radiation would seem to present an excellent opportunity for speciation. As different populations evolve to resemble distinct model species, we expect levels of gene flow between populations to be reduced, and divergence to increase, ultimately leading to speciation. *Heliconius* butterflies provide a clear example of a mimetic radiation where a trait (wing color pattern) is under divergent selection in the context of mimicry and has apparently led to population divergence and speciation. These butterflies are involved in complex Müllerian mimicry rings throughout much of South and Central America [4]. When a single species participates in multiple mimicry rings (i.e. distinct geographic ‘races’), divergence occurs as different populations diverge to conform to their respective mimetic race [12]. This is thought to create a scenario where hybrids have (1) low mating prospects (due to lack of recognition [13], [14], and (2) low survival prospects (where hybrids are no longer afforded the protective benefits of Müllerian mimicry), ultimately leading to divergence and speciation [15].

Here we investigate phenotypic and genetic divergence among populations of the mimic poison frog, *R. imitator*. We expand on previous analyses of diversification in this species, describing a fourth major mimetic morph in addition to the three already described [5], [10], [16], and incorporate analyses of spectral reflectance and melanistic pattern to document divergence in color and pattern. These data reveal the presence of intermediate pattern morphs in “transition-zones” between some of the distinct morphs. We use Bayesian methods of population genetic analysis of microsatellite variation to estimate genetic structure and both recent and historical levels of gene flow among populations, in order to compare them [17]. On the basis of our results, we argue that the levels of divergence among populations are variable, and incomplete or breaking down in some regions. We speculate that this trend may be driven by the ecological dominance of the mimic poison frog associated with its unique reproductive strategy of biparental care. We present data on the relative abundance of the mimic poison frog in relation to its putative models that is consistent with this hypothesis.

## Materials and Methods

### 1. Sampling for Color and Pattern Analyses

**Figure 1:**
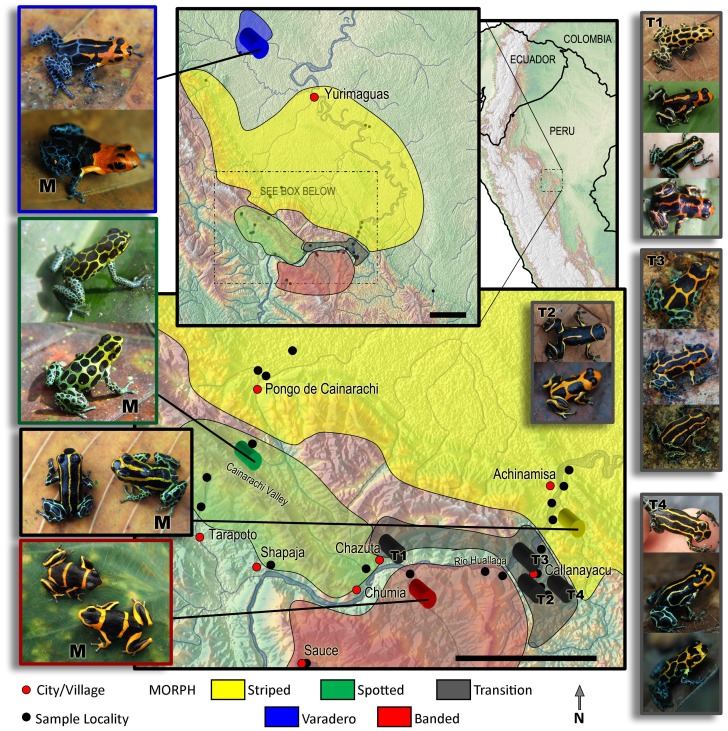
Map showing the four main color patterns and their putative models (left) and highly variable populations (right), in San Martin and Loreto provinces, Peru. Photos on left-hand side show the four mimetic morphs of *R. imitator* and corresponding model species (denoted with “M”). Photos on right-hand side give examples of four transition zones (T1–T4) Black scale bars = 30 km.

*Ranitomeya imitator* individuals from four distinct color pattern morphs from 14 locations throughout the species’ range were sampled from 2002–2008 (Fig. 1, S1; Table S1). Locations were as follows: Tarapoto, Cainarachi Valley, Chumia, Shapaja, Chazuta, Sauce, Chipaota, Curiyacu, Callanayacu, Ricardo Palma, Aguas Termales, Achinamisa and Pongo de Cainarachi (all in San Martin province) and Varadero (Loreto province). The Varadero population represents a fourth mimetic morph and is mimetic with *Ranitomeya fantastica*. Samples from Tarapoto and the Cainarachi Valley were combined for the gene flow analyses, as were the Chumia and Shapaja samples, the Chipaota and Curiyacu samples, and the Ricardo Palma, Aguas Termales and Achinamisa samples. Frogs from these grouped localities were geographically proximate, and were not differentiated phenotypically or genetically (see below).

Phenotypic sampling for this study consisted of dorsal and ventral photographs using a Canon 10d DSLR camera with a 100 mm macro lens and a macro ring flash, in a standardized position, measurement of spectral reflectance with an Ocean Optics Spectrometer (for some samples), and tissue collection in the form of toe clips. In a previous analysis, we used discriminant function analyses to demonstrate that melanistic pattern elements and color differed significantly between four distinct mimetic morphs [8]. Here we repeat these analyses with a wider range of population samples including populations of intermediate morphology (particularly Callanayacu and Chazuta) in the analyses of pattern.

### 2. Pattern

Pattern was analyzed by using ImageJ64 software [18] to investigate variation among populations in the distinct melanistic elements of the color pattern (spots, stripes, bands). Stripes extending from anterior to posterior were classified as ‘stripes’ and those that were perpendicular, extending dorsal-ventrally, as ‘bands’. All dorsal pattern markings were first counted and those that were clearly visible were measured for length, width and area for eight populations (Tarapoto, Cainarachi Valley, Central Huallaga (Curiyacu and Chipaota), Callanayacu, Chazuta, Sauce, Pongo de Cainarachi and Varadero). Using the tracing tool the black regions were traced and the tracings smoothed, and the area calculated. Averages were taken for length, width and area of pattern elements for each individual.

### 3. Spectral Reflectance (color)

We measured spectral reflectance from *R. imitator* frogs across 9 populations: Striped Morph Populations (Pongo de Cainarachi, n = 10 frogs, Aguas Termales n = 1, Ricardo Palma n = 7); Spotted Populations (Cainarachi Valley n = 10 frogs; Tarapoto n = 8); Banded Populations (Curiyacu n = 4; Chipaota n = 4; Sauce, n = 9), and the Varadero Morph (Varadero, n = 10 frogs).

In our analyses, we used reflectance measurements from the head, mid dorsum, lower dorsum and legs (left and right side measurements for each of these areas). Reflectances were evaluated using an avian visual model (e.g. [19]) and the resulting brightness and color contrast estimates served as inputs into a linear Discriminant Function Analyses in R (CANDISC and MASS packages) to determine whether the different morphs (Striped, Banded, Spotted or Varadero) are statistically distinct on the basis of parameters relevant to avian sensitivities (brightness or color contrast), representing a potential tetrachromatic predator. Generalized canonical discriminant analysis allowed us to transform our multidimensional color parameters (color and brightness contrast estimates for the different body regions of the frog) into a canonical space. We used the CANDISC package in R [20], [21] to compute the canonical scores and vectors for the Morph term in our multivariate linear model and used a Type II MANOVA test to test the multivariate linear model. We used the MASS package [22] in R to determine whether the morph classification can be predicted based on the within-class covariance matrix of our multivariate linear model.

### 4. Genetic Structure and Gene Flow

Tissue samples (toe tips) from 166 individuals from the main localities (Table S1, Fig. S1) were collected between 2002–2008, and preserved in vials with 20% DMSO saturated with NaCl. DNA was extracted with the Qiagen Dneasy Tissue Kit (Qiagen Inc, Valencia, CA, USA). Amplification of DNA was done with the polymerase chain reaction (PCR). Gel electrophoresis confirmed successful amplification and samples were cleaned using ExoSap-it™ using standard protocols. Cleaned PCR products were then added for sequencing reactions and cleaned again using hydrated Sephadex™ in Millipore™ plates. Fragment sizes were determined using an ABI 3130 automated sequencer, and analyzed using the ABI Genotyper software (please see [23] for the details of the molecular methods). We used nine polymorphic microsatellites previously developed specifically for *R. imitator* or (in one case) the congeneric *R. variabilis* [24]. Tests for Hardy-Weinberg equilibrium and linkage disequilibrium were carried out with GENEPOP 4.0 [25].

Deciding what constitutes a biologically meaningful population is a challenging and unresolved question, and the answer may differ depending on whether one is interested primarily in ecological or evolutionary phenomena [26]. To address this issue from an evolutionary genetic perspective, we used the program STRUCTURE version 2.3.2 [27]–[29] to infer the genetic structure of the samples. STRUCTURE employs Markov Monte Carlo Sampling in a Bayesian statistical framework to infer genetic structure based on a maximum likelihood model of multilocus genetic equilibrium. We employed the admixture model with linkage allowed. For the first runs, no a priori information on color pattern morph was provided. We used a burnin of 50000 MCMC iterations, and 1000000 iterations after burnin. The program was run ten times for each value of K (population number) from 1–10 (preliminary runs indicated that K would be within this range). These runs showed no clear structure across localities, with the exception of two: Sauce and Varadero, which came out as genetically distinct (data not shown).

On this basis, we classified localities into populations using three criteria: the first results from STRUCTURE (placing Sauce and Varadero into separate populations); color pattern morph (spotted, banded, striped, the Varadero morph, and two intermediate morphs: Chazuta (spotted/banded) and Callanayacu (banded/striped)); and distance (two widely separated localities of the same color pattern morph were designated as distinct populations: Chumia/Shapaja and Tarapoto/Cainarachi Valley; Ricardo Palma/Aguas Termales/Achinamisa and Pongo). In order to maximize the potential to detect genetic differentiation among putative populations, we removed samples from the dataset that did not fall clearly into one or the other of the populations designated above. This affected only the samples between the Chipaota/Curiyacu and Callanayacu populations. This removal was conservative with respect to the results of later analyses, because these samples were not phenotypically or genetically differentiated from each other. We used the LOCPRIOR model to implement the population designations described above as prior information in the model. We then re-ran the analyses (as described above), but using only K = 1–8. We chose the most likely number of genetically distinct populations (K) based on the posterior probabilities for each value of K (ln Pr(*X*/*K*), as described in Pritchard et al. [27].

To estimate recent levels of gene flow between populations, we used the program BAYESASS, which implements a Bayesian method of estimation [30]. It uses transient levels of linkage disequilibrium produced by recent migrants or their immediate descendants to infer levels of migration into populations. The program uses MCMC sampling in a Bayesian statistical framework to estimate gene flow in the recent past (i.e. over the last few generations). Preliminary runs were used to determine the optimal settings. In particular, levels of the delta parameters for allele frequency, inbreeding and migration rate were set so that the proposed changes were approximately 40 to 60% of the total number of iterations. The final run involved 3.0×10^7^ MCMC iterations, with a burn-in of 9.9×10^6^ iterations. The program estimates the mean value for migration rate, and a 95% confidence interval for the estimate. The estimated values can be compared to the expected value and confidence interval when there is no information in the data.

To estimate historical patterns of gene flow, we used the program MIGRATE [31]. This program implements a coalescent genealogical sampling scheme in a maximum likelihood or Bayesian statistical framework to estimate historical migration rates (*M* = *m*/*μ*), where *m* = the migration rate and *μ* = the mutation rate, and Theta (*Θ* = 4*N_e_μ*; where *N_e_* = effective population size). To estimate the migration rate (m), we multiplied M by an estimated mutation rate of 5×10^−4^ [32], allowing comparisons with the value of m estimated by BAYESASS [17]. We used the Bayesian framework, with uniform distributions for the priors. One long chain and four “heated” chains were used to explore the surface of the genealogy and parameter hyperspace, using both “adaptive” and “static” heating schemes on alternate runs. Long chains were run for 2.5×10^6^ MCMC iterations, and the posterior probabilities were sampled every 100 iterations. Once a run was completed, we used the estimated values for Theta and *M* to set the starting values for the next run. This process was continued in an iterative fashion until the program produced consistent results across runs. Given the long period of time taken for each run by this computationally intensive method, and the large number of runs required, we used a reduced dataset that excluded two populations that were shown by the STRUCTURE and BAYESASS analyses (see below) to be genetically isolated and to have low levels of gene flow (Sauce and Varadero). This left us with a total of seven populations for the MIGRATE analyses (Tarapoto/Cainarachi Valley, Chumia/Shapaja, Chazuta, Chipaota/Curiyacu, Callanayacu, Ricardo Palma/Aguas Termales/Achinamisa, and Pongo de Cainarachi).

### 5. Relative Abundance

To estimate relative abundances, we compiled collection records made during the years 2004–2011 on all *Ranitomeya* species in this area. We only included surveys where we had notes on the collection of all species, therefore, collections that focused solely on the capture of *R. imitator* were omitted. During these surveys, frogs were located via visual and acoustic cues. While the model species generally call more quietly and less frequently than *R. imitator*, all *Ranitomeya* species here are detectable using visual surveys [33]. Taxonomic identification was made on the basis of host plant usage, advertisement call structure and adult morphology.

## Results

Our surveys across the range of *R. imitator* revealed the presence of a fourth color pattern mimic, in the Varadero region, that mimics the local model species *R. fantastica*, and appears to be geographically distant from other known *R. imitator* populations (Figure 1). However, our surveys also revealed several regions of contact between distinct color pattern morphs (i.e. banded, striped, spotted), and a large “transition-zone” with intermediate phenotypes (Figure 1, T1–T4). These transition zones are much narrower (roughly 6–10 km wide) than the geographical extent of the ‘pure’ mimetic morphs on either side of the transition zones (ranging from roughly 30–60 km in width). They appear to coincide with a change in the local model species community; for example, the transition zone from the banded to striped morphs occurring near Callanayacu appears to coincide with the distribution of the two local model species (*R. summersi,* present southwest of Callanayacu, and striped *R. variabilis*, present northeast). In the transition zone between the striped and spotted morphs occurring in the foothills south of Pongo de Cainarachi, the color pattern of *R. imitator* closely tracks intraspecific variation in a single, polymorphic model species, *R. variabilis* (previously considered to be two species [34]), which varies along an elevation gradient.

### 1. Pattern Comparisons

Color and pattern differences among the major morphs and their putative models are shown in Figure 1. Representative intermediate morphs from transition zones are also shown in the figure.

**Figure 2:**
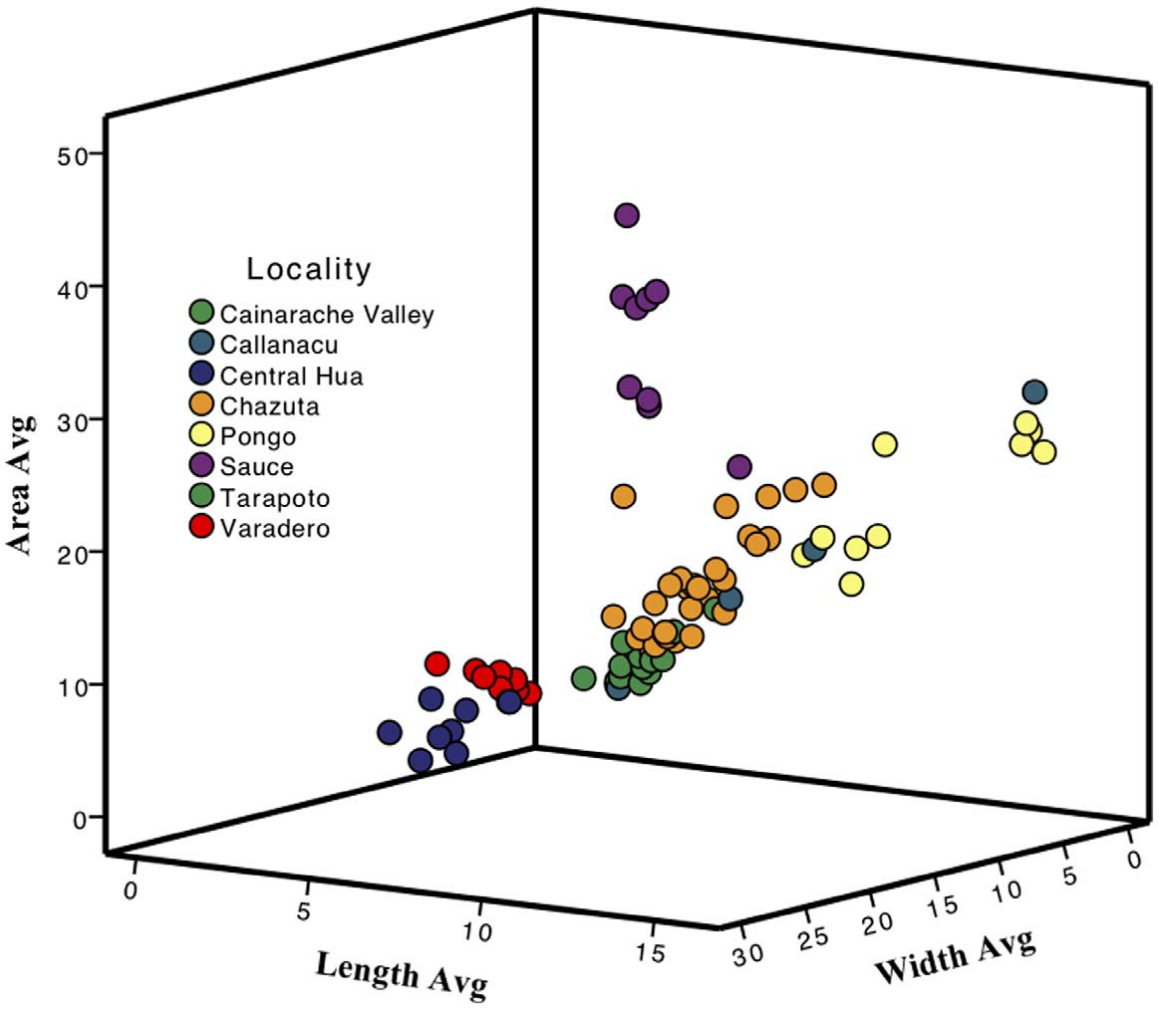
Visual representation of pattern divergence in length, width and area measurements. Populations of phenotypically similar morphs are grouped with matching colored dots.

Figure 2 shows a multidimensional summary of the pattern variation within and between population samples. Analysis of the specific pattern characteristics (length, width and area) revealed distinct differences in melanistic pattern attributes between populations. Length and area explained the highest proportions of the variability in pattern. Both of these characteristics were significantly different (Chi square statistics, p<0.001 for each dimension) between the populations sampled, demonstrating substantial pattern differences between geographic populations (Tables S2, S3). Aspects of black patterning vary significantly between phenotypic types (Fig. 2, Tables S2, S3). These black elements comprise what is commonly referred to as pattern, and makes mimetic similarity possible between species with complex color patterns. Divergence between phenotype groups of *R. imitator* represents pattern divergence as a result of advergence onto model species phenotypes, thus providing enhanced mimetic similarity [35]. Despite the significant levels of pattern divergence, populations from the transition zones show high levels of variation spanning the gaps between major mimetic morphs (Fig. 2). Frogs from Chazuta are typically intermediate between the spotted morph (Tarapoto and the Cainarachi Valley), the banded morph (Sauce) and the striped morph (Pongo de Cainarachi). Frogs from Callanayacu show high levels of variation, with some showing the spotted pattern, some showing the striped pattern, and others being intermediate in color pattern. One unexpected result was that the populations from the central Huallaga region (Chipaota and Curiyacu) were similar in pattern to the frogs from the Varadero region. This similarity is likely due to a cross-shaped break in the black bands that is common to some locations in the Central Huallaga region, disturbing what is otherwise complete black bands. This could result in similarities that are comparable to broken vertical stripes found in some individuals from Varadero.

### 2. Color Comparisons

**Figure 3:**
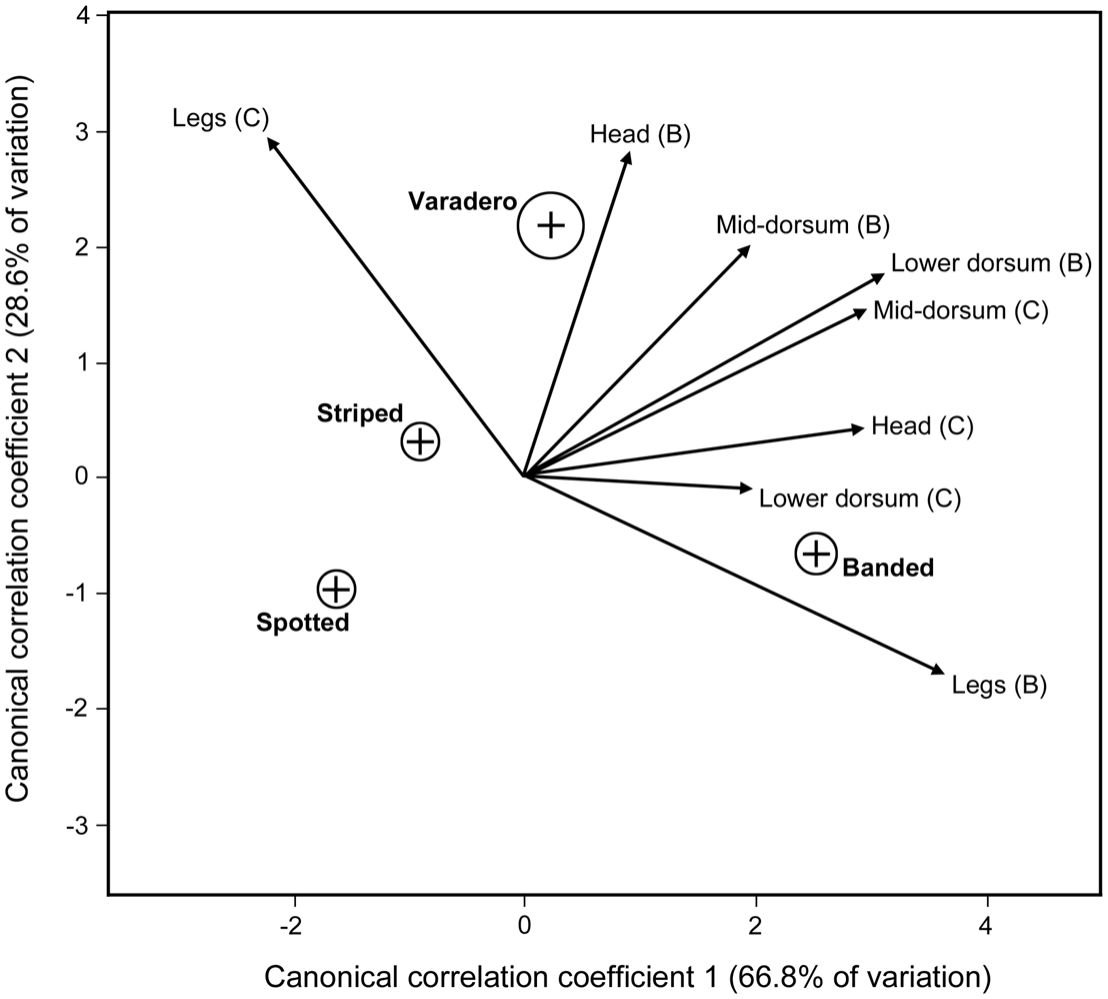
Canonical Space for *R. imitator* morphs using an avian visual model for frog contrast estimates against a green background (*Heliconius* leaf). Crosses represent the average canonical scores and the circles represent the 95% confidence ellipses for the four *R. imitator* morphs. The multivariate linear model included avian model estimates for brightness (B) and color contrast (C) of four body region reflectances (head, mid and lower dorsum, legs).

The discriminant analysis for frog morph by avian viewer produced three significantly different canonical dimensions (Type II MANOVA Wilks statistic = 16.88, p<0.001) with the first canonical axis accounting for 66.8% of the variance, the second canonical axis accounting for 28.6% and the third axis 4.6%. Figure 3 shows the mean canonical scores and 95% confidence ellipses for each morph within the canonical space along with the vectors for each term in the model. The discriminant function analysis was able to correctly assign 87% of all reflectances to appropriate morph classification, with the best performance for the banded morph (94% Banded, 86% Striped, 85% Varadero, and 83% Spotted correct assignment).

### 3. Genetic Analyses

The results of the tests for Hardy-Weinberg Equilibrium and linkage disequilibrium for the microsatellites are shown in Table S4, S5. Two loci (RimiE02 and RimiB01) were found to be out of Hardy-Weinberg equilibrium for most populations using the population HWE tests implemented in GENEPOP. One pair of loci (RimiA06 and RimiB02) showed significant linkage disequilibrium across populations. In order to ensure that this did not affect our results, we re-ran the STRUCTURE analyses without the loci that were not in Hardy-Weinberg equilibrium. We also ran the BAYESASS and MIGRATE analyses without one of the loci in disequilibrium: locus A06 (the algorithms used in these programs do not assume HWE). In each case, the results analyses with the loci removed were almost identical to those from the analyses using the full dataset (data not shown).

**Figure 4:**
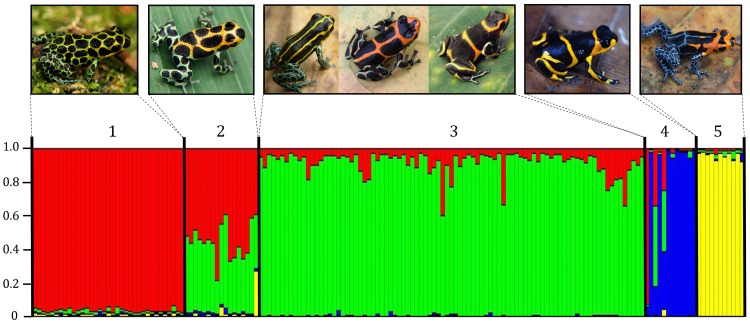
Genetic structure as inferred with the program STRUCTURE (K = 4). Different colors represent inferred multilocus genotypes (see text). Clusters are as follows: 1) Chumia/Shapaja/Tarapoto/Cainarachi Valley, 2) Chazuta, 3) Chipaota/Curiyacu/Callanayacu/Ricardo Palma/Achinamisa/Aguas Termales, 4) Sauce, 5) Varadero. Each individual frog is represented by a vertical line, which is partitioned into *K* +1 segments, with each color representing the individual’s membership coefficient to that group. Chazuta is presented as a separate population in this figure, as it shows a clearly intermediate genotype, but in the optimal grouping derived from STRUCTURE Chazuta is part of cluster 1 (Chumia/Shapaja/Tarapoto/Cainarachi Valley).

An analysis of genetic structure using STRUCTURE with prior information on location and color pattern morph indicated a maximum likelihood value for the number of genetically distinct subgroups (K) of four (Figure 4). The results with the strongest support grouped the Chazuta population (shown as cluster two in the figure) with cluster one (Tarapoto, Cainarachi Valley, Chumia, Shapaja). However, Chazuta appears clearly intermediate in genetic composition between Chumia/Shapaja and Curiyacu/Chipaota (Figure 4), and so we treat it as a distinct population that is genetically and phenotypically intermediate. This allows us to investigate recent and historic levels of gene flow between this region and others in subsequent analyses. The distinct clusters consisted of the following localities (and associated morphs): 1) Tarapoto, Cainarachi Valley, Chumia and Shapaja (spotted); 2) Chazuta (intermediate spotted/banded); 3) Chipaota, Curiyacu (banded), Callanayacu (intermediate banded/striped), Ricardo Palma, Aguas Termales, Achinamisa, Pongo de Cainarachi (striped); 4) Sauce (banded); 5) Varadero (Varadero morph). As noted in the introduction, populations between Curiyacu/Chipaota and Callanayacu showed no genetic differentiation and were omitted from the final STRUCTURE analyses. The lack of genetic differentiation among these populations is fully consistent with the lack of differentiation seen across more widely separated populations in this region (included populations that differ dramatically in color pattern).

Analyses of recent gene flow using BAYESASS yielded patterns that were generally consistent with the genetic clusters inferred using STRUCTURE. Table 1 shows the estimated mean migration rates (see Table S6 for the 95% confidence intervals) for each pairwise comparison between populations, as defined using a specific combination of color pattern morph and geographical isolation (see methods). Note that levels of gene flow lower than 0.126 are not informative (i.e. cannot be distinguished from random noise) using this method with this dataset, and this was the case for most comparisons in this study.

**Table 1:** Rate of recent gene flow (*m*) derived from the program BAYESASS (Wilson & Ranala 2003).

	Tara	Chum	Chaz	Sauce	Curiy	Callan	Achin	Pongo	Vara
**Tarapoto**	–	0.004	0.015	0.016	0.002	0.022	0.006	0.031	0.005
**Chumia**	**0.173**	–	**0.166**	0.007	0.003	0.009	0.01	**0.136**	0.005
**Chazuta**	0.006	0.003	–	0.005	0.002	0.008	0.006	0.014	0.004
**Sauce**	0.005	0.003	0.026	–	0.002	0.01	0.006	0.015	0.004
**Curiyacu**	0.005	0.004	0.067	0.006	–	**0.246**	**0.275**	0.068	0.007
**Callanayacu**	0.003	0.003	0.009	0.006	0.002	–	0.006	0.014	0.004
**Achinamisa**	0.003	0.003	0.009	0.005	0.002	0.008	–	0.014	0.004
**Pongo**	0.003	0.003	0.009	0.006	0.002	0.008	0.006	–	0.004
**Varadero**	0.003	0.003	0.014	0.005	0.002	0.008	0.006	0.014	–

Results are given as the level of gene flow (proportion migrants per generation) from the population on the left (row headings) into the populations on the right (column headings). Bolded values indicate rates that can be statistically distinguished from non-informative data.

There are high levels of recent gene flow from Chumia/Shapaja into Tarapoto/Cainarachi Valley, in spite of the fact that these localities are a considerable distance apart (approximately 19 km between the nearest locations). However, frogs from all of these locations are spotted and occur at similar elevations, so this result is not surprising.

There is also a high level of gene flow from Chumia/Shapaja into Chazuta. Interestingly, frogs in Chazuta are also intermediate in appearance between the spotted and banded morphs (Figure 1). Therefore, this site appears to represent a transition zone between the two morphs. There is also a somewhat elevated estimate of gene flow from Chumia/Shapaja into Pongo, which was an unexpected result. The highest levels of gene flow are found from the Curiyacu/Chipaota population into the Callanayacu and Ricardo Palma/Aguas Termales/Achinamisa populations. The Curiyacu/Chipaota population is banded, the Callanayacu population is mixed banded/striped, and the Ricardo Palma/Aguas Termales/Achinamisa population is striped. The high levels of gene flow from Curiyacu/Chipaota into both of these populations is consistent with the lack of genetic structure among these populations revealed by the STRUCTURE analyses (Fig. 4). Note that the results from the BAYESASS analyses generally show significant gene flow in one direction only. This may reflect general patterns of introgression mediated by ease of dispersal (e.g. downstream in the case of the Huallaga Canyon), but this requires further investigation.

The results of the MIGRATE analysis are shown in Table 2. The results show low levels of historical (long-term average) gene flow among most populations. There is no obvious tendency for gene flow to be higher between populations that have the same color pattern morph relative to pairs that have different morphs. In fact, the mean pairwise estimate of gene flow between populations of similar morphs is slightly lower (0.014) than mean pairwise estimates of gene flow between populations differing in morph (0.016), but these differences are not significant. These results are consistent with the hypothesis that some of the populations containing different color pattern morphs were more genetically isolated in the past than they are now.

**Table 2:** Rates of historical gene flow (*m*) derived from the program MIGRATE (Beerli & Felsenstein 1999).

	Tara	Chum	Chaz	Curiy	Callan	Achin	Pongo
**Tarapoto**	**0.098**	0.013	0.012	0.018	0.015	0.015	0.016
**Chumia**	0.008	**0.098**	0.013	0.01	0.014	0.011	0.014
**Chazuta**	0.016	0.015	**0.098**	0.009	0.015	0.019	0.012
**Curiyacu**	0.014	0.017	0.012	**0.098**	0.014	0.018	0.014
**Callanayacu**	0.016	0.016	0.013	0.011	**0.098**	0.012	0.025
**Achinamisa**	0.02	0.013	0.017	0.018	0.016	**0.098**	0.015
**Pongo**	0.011	0.012	0.011	0.01	0.01	0.013	**0.097**

Bolded values down the diagonal represent estimates of Theta (*θ*) for each population. Results are given as the level of gene flow (proportion migrants per generation) from the population on the left (row headings) into the populations on the right (column headings).

**Figure 5:**
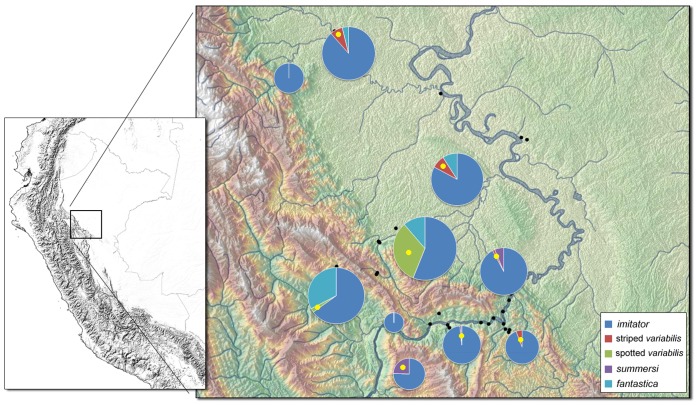
Relative abundance of *R. imitator* vs. other sympatric species of *Ranitomeya*. The yellow dot indicates which species is the putative model in that site. These data were obtained from sampling trips over the years 2004–2010 based primarily on field notes, photographic records, and mark-recapture surveys made by current and former lab members. Overall, 791 frogs were recorded, 671 of which were *R. imitator*.

In our surveys, we collected all small poison frogs encountered in field surveys (both *R. imitator* and the putative model species), allowing us to estimate the relative abundance of the mimic and its models. We consistently found higher numbers of *R. imitator* compared to the model species in all areas. In each locality, *R. imitator* was substantially more common than any other congeneric species (Figure 5). See Table S7 for raw counts of each species at each sampling locality.

## Discussion

Our phenotypic analyses indicate that the different mimetic morphs are significantly different from one another (see also [8]) both with respect to aspects of black patterning (Fig. 2) and dorsal coloration (Fig. 3). Using an avian visual model to estimate the contrast of the frogs for both brightness and color (hue), we found that the different morphs are significantly different using Discriminant Function Analysis (Fig. 3).

Our survey of color pattern phenotypes across populations indicates that in the transition zone involving contact between distinct morphs (with Chazuta at the west end and Callayanacu at the east end: see Fig. 1 and Fig. S1) there are populations that exhibit color patterns intermediate between those of either morph. There are two potential explanations for the presence of intermediate phenotypes in these transition areas. One is that they represent offspring from matings between different mimetic morphs. Another possibility would be selection for intermediate forms. These intermediate forms do not appear to closely resemble either model species, so selection for these forms seems unlikely. We interpret this as evidence of mixing across morph boundaries.

Our analysis of genetic structure demonstrates that the only strongly divergent populations are Sauce and Varadero. Both of these populations are peripheral, and Varadero is geographically distant from the other populations. Sauce is also separated from other populations by mountainous terrain (Fig. S1). More sensitive analyses (incorporating prior information on mimetic morphs – see materials and methods) reveal some structure between the spotted morph populations and the banded and striped morph populations (with the Chazuta population showing clearly intermediate genotypes, consistent with its color pattern phenotype). The banded and striped populations (with the exception of Sauce) show no evidence of genetic discontinuity. The analysis of recent patterns of gene flow were consistent with the results of the genetic structure analysis, and indicated substantial gene flow between populations with distinct color pattern morphs, particularly among the Chipaota and Curiyacu (banded), Callanayacu (mixed banded/striped) and the Ricardo Palma, Aguas Termales and Achinamisa (striped) populations, all of which clustered into a single group in the analysis of genetic structure. As noted in the introduction and results, populations from the regions between Chipaota/Curiyacu and Callanayacu also showed no genetic structure.

Previous analysis demonstrated strong phenotypic (pattern) resemblance between *R. imitator* from banded populations (Sauce, Chipaota and Curiyacu), spotted populations (Tarapoto/Cainarachi Valley and Chumia/Shapaja), striped populations (Ricardo Palma, Aguas Termales and Achinamisa) and putative model species from each area [35]. Under a scenario with strong correspondence between mimetic morph and genetic group, we would have expected the banded frogs in the Huallaga river canyon (Chipaota and Curiyacu) to fall in the same genetic cluster as the banded frogs from Sauce, but they do not. Similarly, we would have expected a genetic gap between the striped populations (Ricardo Palma, Aguas Termales and Achinamisa) and the banded populations from Chipaota and Curiyacu, but there is no genetic structure separating these populations. Furthermore, frogs originating from the transition zone between the banded and striped populations (for example, near Callanayacu) show wide variation in phenotype, with some individuals clearly intermediate between the two morphs (Fig. 2). Overall, these results are suggestive of mimetic and genetic divergence in peripheral populations, and mixing in more central populations.

Overall, our comparisons of historical versus contemporary estimates of gene flow suggest that gene flow was lower in the past than it is now in several regions. Therefore, rather than indicating that population divergence is stable or escalating over time, this suggests that some of the different mimetic morphs may be in the process of fusing. Given the likelihood that mimicry initially led to strong morphological divergence between populations [5], [8], [10], [16], why does divergence currently appear to be breaking down in some regions?

One possibility is a lack of model species in the regions where the intermediate morphs occur. However, while we have not found potential model species in some regions that *R. imitator* inhabits, in most of the areas where intermediate morphs occur model species are present. Hence this explanation seems unlikely.

Another possibility is that there has been an interaction between the unique reproductive strategy of *R. imitator* and the dynamics of mimicry evolution. Although all dendrobatids show parental care, *R. imitator* is unusual in that it shows biparental care and trophic egg-feeding. Tadpoles are deposited in very small pools in plant leaf axils (phytotelmata), and the male and female cooperate to care for the tadpoles during development. The female provides infertile eggs for the tadpole to eat until it reaches metamorphosis [33]. This reproductive strategy results in a high growth rate for tadpoles in this species relative to at least one of the sympatric model species, *R. variabilis* [23]. The higher availability and density of the smaller pools that *R. imitator* are able to utilize, relative to the sporadically located larger pools used by the putative model species also likely contributes to higher population densities. Finally, in most cases several non-egg feeding species are present, and compete for access to larger pools (such as treeholes and bromeliads), further limiting the population densities of the model species.

As described above, *R. imitator* consistently shows higher abundance than the model species with which it co-occurs (Fig. 5). This result suggests the following hypothesis. When *R. imitator* first colonized the region of San Martin and Loreto provinces it currently occupies, it was a new immigrant and hence likely to be rare relative to the other species of dendrobatid frogs already occupying those regions. Theory suggests that the rare species in a pair of aposematically-colored, chemically defended species will be under substantially stronger selection to “adverge” on the color pattern of the common species rather than the reverse in systems of Müllerian mimicry [1], [2]. This is because local predators typically learn to recognize and avoid the most common local color pattern. Hence, during the early period of colonization by *R. imitator*, it, rather than the local model species, was probably strongly selected to adverge on the most common color pattern. This likely drove the initial divergence among morphs that we currently see between many *R. imitator* populations [5], [8], [10], [16]. Note that this hypothesis assumes that *R. imitator* remained at relatively low population density after colonizing the north for a period long enough to allow selection to change color pattern across populations.

Once *R. imitator* became established, its unique reproductive strategy apparently allowed it to achieve population densities that were substantially higher than those of the local model species. In turn, this should cause selection on mimicry in *R. imitator* populations to relax. Essentially, these populations would become dense enough that *R. imitator* would become the most common poison frog encountered by local predators, and hence would largely be responsible for “training” those predators. Ultimately, this should result in predators learning to avoid all morphs of *R. imitator* (or to have a more generalized avoidance response) as different morphs become more frequently encountered with increasing population density.

This hypothesis is speculative, but makes specific predictions that can be tested. It predicts that predators occurring in areas where *R. imitator* is highly variable (i.e., mimetic transition zones) will avoid a wider range of aposematic phenotypes than in areas where *R. imitator* phenotypes are ‘fixed’ on a single mimetic morph. This hypothesis also predicts that the “classic” mimetic morphs and the intermediate morphs in the transition zones at Chazuta and Callanayacu will show similar levels of protection. These predictions are currently under investigation.

There are, of course, alternative (non-mutually exclusive) hypotheses that could explain a breakdown in divergence across *R. imitator* populations. For example, it is possible that *R. imitator* is actually more toxic than the model species in this system. Again, if *R. imitator* populations were initially at low density relative to those of the model species, then it would be under selection to adverge on the models in spite of its higher toxicity, for reasons discussed above. However, once *R. imitator* reached substantial population densities, such selection should be relaxed, and fusion might occur. Currently, we know that both *R. imitator* and its model species are toxic [36], but relative levels of toxicity are not known at this time.

Activity levels could also be involved. For example, if *R. imitator* is more likely to be active during the day than a model species like *R. variabilis*, it might come to play a more pronounced role in predator education once it becomes established. However, we have done extensive behavioral observations on these species (e.g. [33]), and have not observed differences that would support the predictions of this hypothesis.

## Supporting Information

Figure S1 **Map of all sampling localities mentioned in the text. Black scale bar equals 20 km.** Two localities (Balsapuerto and Chipesa) were used only in the relative abundance analysis.
(TIFF)

Table S1 **Localities for collection of samples in this study, in San Martin and Loreto Province, Peru.**
(DOCX)

Table S2 **Significance tests of the discriminant dimensions (see text).**
(DOCX)

Table S3 **Standardized discriminant coefficients for each dimension (see text).**
(DOCX)

Table S4 **Tests for violations of Hardy-Weinberg Equilibrium for each locus across all populations.**
(DOCX)

Table S5 **Tests for linkage disequilibrium between microsatellite loci.**
(DOCX)

Table S6 **Levels of gene flow estimated with BayesAss, including 95% confidence intervals.** The site “Pongo de Cainarachi” as shown on Fig. S1 has been shortened to “Pongo”.
(DOCX)

Table S7 **Raw counts for relative abundance analysis.** Numbers refer to the number of individuals found for that species in that site. A zero is given for cases where that species is assumed to occur at that site but was never found. Blank fields indicate the species is likely absent from that site. The superscript “m” indicates the putative model species at a given site. For the two transition zone populations, no model species is specified because the *R. imitator* phenotypes do not necessarily correspond to any model species.
(DOCX)

## References

[1] Ruxton G, Sheratt T, Speed M (2004) Avoiding Attack: The Evolutionary Ecology of Crypsis, Warning Signals and Mimicry. Oxford: Oxford University.

[2] Müller F (1879) *Ituna* and *Thyridia*; a remarkable case of mimicry in butterflies. Trans Entomol Soc Lond 1879: xx–xxix.

[3] Rowland HM, Hoogesteger T, Ruxton GD, Speed MP, Mappes J (2010) A tale of 2 signals: signal mimicry between aposematic species enhances predator avoidance learning. Behav Ecol 21: 851–860.

[4] Mallet J, Joron M (1999) Evolution of diversity in warning color and mimicry: Polymorphisms, shifting balance, and speciation. Ann Rev Ecol Syst 30: 201–233.

[5] Symula R, Schulte R, Summers K (2001) Molecular phylogenetic evidence for a mimetic radiation in Peruvian poison frogs supports a Mullerian mimicry hypothesis. Proc Roy Soc Lond B 268: 2415–2421. 10.1098/rspb.2001.1812 PMC1088895 11747559

[6] Mallet J, Barton NH (1989) Strong natural selection in a warning-color hybrid zone. Evolution 43: 421–431. 28568556 10.1111/j.1558-5646.1989.tb04237.x

[7] Chouteau M, Angers B (2011) The role of predation in maintaining the geographic structure of aposematic signals. Am Nat 178: 810–817. 22089874 10.1086/662667

[8] Yeager J, Brown JL, Morales V, Cummings M, Summers K (2012) Testing for selection on color and pattern in a mimetic radiation. Curr Zool 58: 668–676.

[9] Chouteau M, Summers K, Morales V, Angers B (2011) Advergence in Mullerian mimicry: the case of the poison dart frogs of northern Peru revisited. Biol Lett 7: 796–800. 21411452 10.1098/rsbl.2011.0039 PMC3169040

[10] Symula R, Schulte R, Summers K (2003) Molecular systematics and phylogeography of Amazonian poison frogs of the genus Dendrobates. Mol Phyl Evol 26: 452–475. 10.1016/s1055-7903(02)00367-6 12644404

[11] Mallet J (2001) Causes and consequences of a lack of coevolution in Müllerian mimicry. Evol Ecol 13: 777–806.

[12] Jiggins CD, Naisbit RE, Coe RL, Mallet J (2001) Reproductive isolation caused by color pattern mimicry. Nature 411: 302–305. 11357131 10.1038/35077075

[13] Naisbit RE, Jiggins CD, Mallet J (2001) Disruptive sexual selection against hybrids contributes to speciation between *Heliconius cydno* and *Heliconius melpomene.* . Proc Roy Soc Lond B 268: 1849–1854. 10.1098/rspb.2001.1753 PMC1088818 11522205

[14] Kronforst MR, Young LG, Gilbert LE (2007) Reinforcement of mate preferences among hybridizing *Heliconius* butterflies. J Evol Biol 20: 278–285. 17210020 10.1111/j.1420-9101.2006.01198.x

[15] Jiggins C (2008) Ecological speciation in mimetic butterflies. BioScience 58: 541–548.

[16] Schulte R (1999) *Die Pfeilgiftfrösche Vol. II: Peru*. Arteneil, Waiblingen: INIBICO.

[17] Chiucchi JE, Gibbs HL (2010) Similarity of contemporary and historical gene flow among highly fragmented populations of an endangered rattlesnake. Mol Ecol 19: 5345–5358. 20964755 10.1111/j.1365-294X.2010.04860.x

[18] Rasband WS (1997) ImageJ, U. S. National Institutes of Health, Bethesda, Maryland, USA, http://imagej.nih.gov/ij/1997-2011.

[19] Cummings ME, Jordão JM, Cronin TW, Oliveira RF (2008) Visual ecology of the fiddler crab, *Uca tangeri*: effects of sex, viewer and background on conspicuousness. Anim Behav 75: 175–188.

[20] Cooley WW, Lohnes PR (1971) Multivariate Data Analysis. New York: Wiley.

[21] Friendly M (2007) HE plots for Multivariate General Linear Models. J Comp Graph Stat 16: 421–444.

[22] Venables WN, Ripley BD (2002) Modern Applied Statistics with S. Fourth Edition. New York: Springer.

[23] Brown JL, Morales V, Summers K (2010) A key ecological factor drove the evolution of biparental care and monogamy in an amphibian. Amer Nat 175: 436–446. 20180700 10.1086/650727

[24] Brown JL, Chouteau M, Glenn T, Summers K (2009) The development and analyses of twenty-one microsatellite loci for three species of Amazonian poison frogs. Cons Gen Resour 1: 149–151.

[25] Rousset R (2008) GENEPOP’007: a complete re-implementation of GENEPOP software for Windows and Linux. Mol Ecol Resour 8: 103–106. 21585727 10.1111/j.1471-8286.2007.01931.x

[26] Waples RS, Gaggiotti O (2006) What is a population? An empirical evaluation of some genetic methods for identifying the number of gene pools and their degree of connectivity. Mol Ecol 15: 1419–1439. 16629801 10.1111/j.1365-294X.2006.02890.x

[27] Pritchard JK, Stephens M, Donnelly P (2000) Inference of population structure using multilocus genotype data. Genetics 155: 945–959. 10835412 10.1093/genetics/155.2.945 PMC1461096

[28] Falush D, Stephens M, Pritchard JK (2003) Inference of population structure: Extensions to linked loci and correlated allele frequencies. Genetics 164: 1567–1587. 12930761 10.1093/genetics/164.4.1567 PMC1462648

[29] Falush D, Stephens M, Pritchard JK (2007) Inference of population structure using multilocus genotype data: dominant markers and null alleles. Mol Ecol Notes 7: 574–578. 18784791 10.1111/j.1471-8286.2007.01758.x PMC1974779

[30] Wilson GA, Rannala B (2003) Bayesian inference of recent migration rates using multilocus genotypes. Genetics 163: 1177–1191. 12663554 10.1093/genetics/163.3.1177 PMC1462502

[31] Beerli P, Felsenstein J (1999) Maximum-likelihood estimation of migration rates and effective population numbers in two populations using a coalescent approach. Genetics 152: 763–773. 10353916 10.1093/genetics/152.2.763 PMC1460627

[32] Garza JC, Williamson EG (2001) Detection of reduction in population size using data from microsatellite loci. Mol Ecol 10: 305–318. 11298947 10.1046/j.1365-294x.2001.01190.x

[33] Brown JL, Twomey E, Morales V, Summers K (2008) Mating and parental care behaviors in relation to pool use in two species of Peruvian poison frogs. Behaviour 145: 1139–1165.

[34] Brown JL, Twomey E, Amezquita A, Barbosa de Souza M, Caldwell JP, et al (2011) A taxonomic revision of the Neotropical poison frog genus *Ranitomeya* (Amphibia: Dendrobatidae). Zootaxa 3083: 1–120.

[35] Yeager J (2009) Unpublished Masters Thesis, East Carolina University.

[36] Spande TF, Poonam J, Garraffo HM, Pannell LK, Yeh HJC, et al (1999) Occurrence and significance of decahydroquinolines from dendrobatid poison frogs and a myrmicine ant: use of ^1^H and ^13^C NMR in their conformational analysis. J Nat Prod 62: 5–21. 9917275 10.1021/np980298v

