# Supplementary figures and images for: Phenotypic and Genetic Divergence among Poison Frog Populations in a Mimetic Radiation
Source: PLoS One. 2013 Feb 6;8(2):e55443. doi: 10.1371/journal.pone.0055443 (PMC3566184; doi:10.1371/journal.pone.0055443)

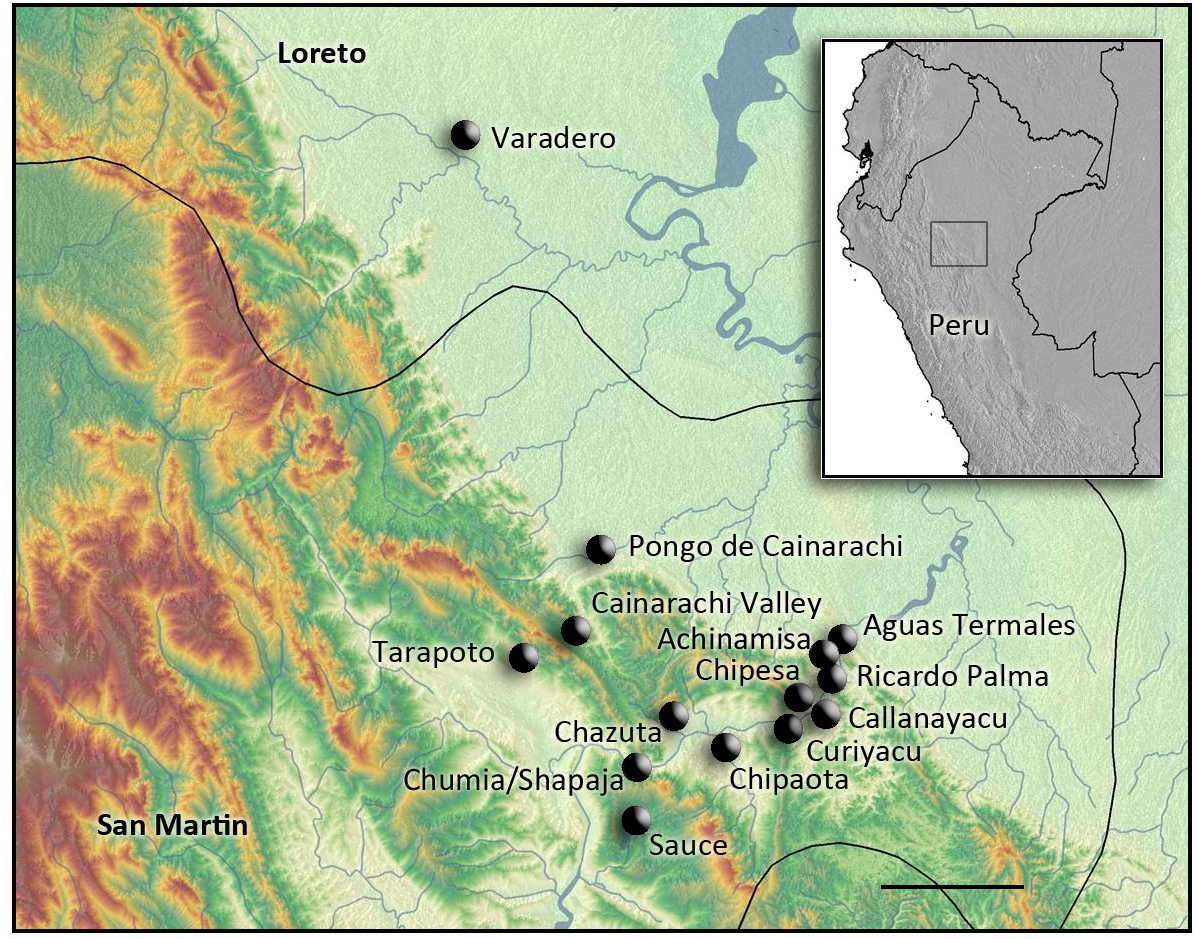

Supplement: Figure S1 — Map of all sampling localities mentioned in the text. Black scale bar equals 20 km. Two localities (Balsapuerto and Chipesa) were used only in the relative abundance analysis. (TIFF) [file pone.0055443.s001.tif]
