# Supplementary material for: Phenotypic and Genetic Divergence among Poison Frog Populations in a Mimetic Radiation
Source: PLoS One. 2013 Feb 6;8(2):e55443. doi: 10.1371/journal.pone.0055443 (PMC3566184; doi:10.1371/journal.pone.0055443)
Supplement: Table S1 — Localities for collection of samples in this study, in San Martin and Loreto Province, Peru. (DOCX) [file pone.0055443.s002.docx]

| Location | Morph | Samples |
| --- | --- | --- |
| Tarapoto/Cainarachi Valley | Spotted | 22 |
| Chumia/Shapaja | Spotted | 13 |
| Chazuta | Mixed | 17 |
| Chipaota/Curiyacu | Banded | 28 |
| Sauce | Banded | 12 |
| Callanayacu | Mixed | 30 |
| Ricardo Palma/Aguas Termales/Achinamisa | Striped | 23 |
| Pongo de Cainarachi | Striped | 10 |
| Varadero | Varadero | 11 |
| Total |  | 166 |
