# Supplementary material for: Phenotypic and Genetic Divergence among Poison Frog Populations in a Mimetic Radiation
Source: PLoS One. 2013 Feb 6;8(2):e55443. doi: 10.1371/journal.pone.0055443 (PMC3566184; doi:10.1371/journal.pone.0055443)
Supplement: Table S2 — Significance tests of the discriminant dimensions (see text). (DOCX) [file pone.0055443.s003.docx]

| Dimension | Canonical Correlation | Chi-square | df | p |
| --- | --- | --- | --- | --- |
| 1 | 0.919 | 320.24 | 15 | 0.00 |
| 2 | 0.831 | 166.33 | 8 | 0.00 |
| 3 | 0.755 | 69.57 | 3 | 0.00 |
|  |  |  |  |  |
