# Supplementary material for: Phenotypic and Genetic Divergence among Poison Frog Populations in a Mimetic Radiation
Source: PLoS One. 2013 Feb 6;8(2):e55443. doi: 10.1371/journal.pone.0055443 (PMC3566184; doi:10.1371/journal.pone.0055443)
Supplement: Table S3 — Standardized discriminant coefficients for each dimension (see text). (DOCX) [file pone.0055443.s004.docx]

| Dimension | 1 | 2 | 3 |
| --- | --- | --- | --- |
| Area | 1.614 | .161 | -.101 |
| Length | -1.361 | .891 | .010 |
| Width | .105 | .298 | .997 |
