# Supplementary material for: Phenotypic and Genetic Divergence among Poison Frog Populations in a Mimetic Radiation
Source: PLoS One. 2013 Feb 6;8(2):e55443. doi: 10.1371/journal.pone.0055443 (PMC3566184; doi:10.1371/journal.pone.0055443)
Supplement: Table S4 — Tests for violations of Hardy-Weinberg Equilibrium for each locus across all populations. (DOCX) [file pone.0055443.s005.docx]

| Locus | Chi Square | df | P-value |
| --- | --- | --- | --- |
| **RimiB07** | 22.92 | 20 | 0.29 |
| **RimiD04** | 5.06 | 16 | 1.00 |
| **RimiE02** | >1000 | 20 | 0.00 |
| **RimiB02** | 22.96 | 20 | 0.29 |
| **RimiC05** | 13.64 | 20 | 0.85 |
| **RimiB01** | >1000 | 20 | 0.00 |
| **RimiF06** | 15.32 | 20 | 0.76 |
| RimiD01 | 13.25 | 20 | 0.87 |
| **RimiA06** | 30.44 | 20 | 0.06 |
