# Supplementary material for: Phenotypic and Genetic Divergence among Poison Frog Populations in a Mimetic Radiation
Source: PLoS One. 2013 Feb 6;8(2):e55443. doi: 10.1371/journal.pone.0055443 (PMC3566184; doi:10.1371/journal.pone.0055443)
Supplement: Table S5 — Tests for linkage disequilibrium between microsatellite loci. (DOCX) [file pone.0055443.s006.docx]

| Locus 1 | Locus 2 | Chi Square | df | P-value |
| --- | --- | --- | --- | --- |
| B07 | D4 | 5.16 | 16 | 0.99 |
| B07 | E02 | 0.00 | 18 | 1.00 |
| D4 | E02 | 8.26 | 18 | 0.97 |
| B07 | B02 | 7.27 | 10 | 0.70 |
| D4 | B02 | 9.76 | 14 | 0.78 |
| E02 | B02 | 19.55 | 14 | 0.15 |
| B07 | C05 | 2.87 | 18 | 1.00 |
| D4 | C05 | 9.96 | 20 | 0.97 |
| E02 | C05 | 15.72 | 18 | 0.61 |
| B02 | C05 | 21.59 | 14 | 0.09 |
| B07 | B01 | 3.44 | 18 | 1.00 |
| D4 | B01 | 14.42 | 20 | 0.81 |
| E02 | B01 | 11.01 | 20 | 0.95 |
| B02 | B01 | 13.52 | 14 | 0.49 |
| C05 | B01 | 10.93 | 18 | 0.90 |
| B07 | F06 | 14.67 | 12 | 0.26 |
| D4 | F06 | 7.35 | 16 | 0.97 |
| E02 | F06 | 17.53 | 16 | 0.35 |
| B02 | F06 | 14.87 | 12 | 0.25 |
| C05 | F06 | 5.20 | 16 | 0.99 |
| B01 | F06 | 14.80 | 16 | 0.54 |
| B07 | D01 | 6.11 | 16 | 0.99 |
| D4 | D01 | 8.94 | 16 | 0.92 |
| E02 | D01 | 5.34 | 16 | 0.99 |
| B02 | D01 | 8.85 | 14 | 0.84 |
| C05 | D01 | 5.32 | 18 | 1.00 |
| B01 | D01 | 16.54 | 16 | 0.42 |
| F06 | D01 | 12.43 | 14 | 0.57 |
| B07 | A06 | 4.42 | 16 | 1.00 |
| D4 | A06 | 12.27 | 18 | 0.83 |
| E02 | A06 | 17.35 | 18 | 0.50 |
| B02 | A06 | >1000 | 14 | 0.00 |
| C05 | A06 | 6.37 | 18 | 0.99 |
| B01 | A06 | 23.69 | 16 | 0.10 |
| F06 | A06 | 12.58 | 16 | 0.70 |
| D01 | A06 | 16.35 | 14 | 0.29 |
