# Supplementary material for: Phenotypic and Genetic Divergence among Poison Frog Populations in a Mimetic Radiation
Source: PLoS One. 2013 Feb 6;8(2):e55443. doi: 10.1371/journal.pone.0055443 (PMC3566184; doi:10.1371/journal.pone.0055443)
Supplement: Table S6 — Levels of gene flow estimated with BayesAss, including 95% confidence intervals. The site “Pongo de Cainarachi” as shown on Fig. S1 has been shortened to “Pongo”. (DOCX) [file pone.0055443.s007.docx]

|  | **Tarapoto** | **Chumia** | **Chazuta** | **Sauce** |
| --- | --- | --- | --- | --- |
| **Tarapoto** | - | 0.004 (1.0e-12-0.03) | 0.015 (1.86e-05-0.06) | 0.016 (8.81e-07-0.07) |
| **Chumia** | 0.173 (0.1-0.31) | - | 0.166 (0.04-0.3) | 0.007 (4.13e-08-0.06) |
| **Chazuta** | 0.006 (4.35e-7-0.03) | 0.003 (8.82e-13-0.03) | - | 0.005 (4.26e-09-0.04) |
| **Sauce** | 0.005 (3.75e-7-0.03) | 0.003 (4.19-3-13-0.02) | 0.026 (5.17e-05-0.08) | - |
| **Curiyacu** | 0.005 (3.75e-7-0.05) | 0.004 (4.56e-12-0.03) | 0.067 (5.13e-05-0.20) | 0.006 (6.65e-09-0.05) |
| **Callanayacu** | 0.003 (1.85e-12-0.03) | 0.003 (1.85e-12-0.03) | 0.009 (1.49e-05-0.04) | 0.006 (2.78e-09-0.04) |
| **Achinamisa** | 0.003 (5.87e-13-0.03) | 0.003 (5.87e-13-0.03) | 0.009 (1.01e-05-0.05) | 0.005 (7.03e-09-0.04) |
| **Pongo** | 0.003 (2.9e-13-0.02) | 0.003 (2.9e-13-0.02) | 0.009 (1.47e-05-0.05) | 0.006 (4.83e-09-0.04) |
| **Varadero** | 0.003 (2.26e-13-0.03) | 0.003 (2.26e-13-0.03) | 0.014 (4.57e-05-0.06) | 0.005 (7.82e-09-0.04) |
|  |  |  |  |  |
|  | **Curiyacu** | **Callanayacu** | **Achinamisa** | **Pongo** |
| **Tarapoto** | 0.002 (1.29e-12-0.02) | 0.022 (4.49e-06-0.07) | 0.006 (3.72e-07-0.03) | 0.031 (0.0002-0.08) |
| **Chumia** | 0.003 (5.72e-13-0.02) | 0.009 (1.56e-6-0.05) | 0.01 (7.56e-07-0.05) | 0.136 (0.05-0.26) |
| **Chazuta** | 0.002 (5.87e-13-0.02) | 0.008 (1.17e-06-0.04) | 0.006 (2.12e-07-0.03) | 0.014 (5.95e-05-0.06) |
| **Sauce** | 0.002 (2.31e-13-0.16) | 0.01 (1.76e-06-0.05) | 0.006 (2.88e-07-0.03) | 0.015 (8.09e-05-0.061) |
| **Curiyacu** | - | 0.246 (0.16-0.32) | 0.275 (0.20-0.32) | 0.068 (0.01-0.17) |
| **Callanayacu** | 0.002 (4.09e-13-0.02) | - | 0.006 (2.83e-07-0.33) | 0.014 (1.00e-03-0.06) |
| **Achinimisa** | 0.002 (9.91e-13-0.02) | 0.008 (1.59e-06-0.04) | - | 0.014 (8.73e-05-0.06) |
| **Pongo** | 0.002 (2.23e-12-0.02) | 0.008 (1.03e-06-0.04) | 0.006 (2.83e-07-0.03) | - |
| **Varadero** | 0.002 (7.98e-13-0.02) | 0.008 (1.03e-06-0.04) | 0.006 (2.69e-07-0.03) | 0.014 (8.46e-05-0.06) |
|  |  |  |  |  |
|  | **Varadero** |  |  |  |
| **Tarapoto** | 0.005 (4.62e-12-0.03) | |  |  |
| **Chumia** | 0.005 (8.54e-12-0.04) | |  |  |
| **Chazuta** | 0.004 (6.61e-12-0.04) | |  |  |
| **Sauce** | 0.004 (1.28e-11-0.04) | |  |  |
| **Curiyacu** | 0.007 (1.85e-11-0.05) | |  |  |
| **Callanayacu** | 0.004 (7.69e-12-0.03) | |  |  |
| **Achinamisa** | 0.004 (9.53e-12-0.04) | |  |  |
| **Pongo** | 0.004 (8.89e-12-0.04) | |  |  |
| **Varadero** | - |  |  |  |
