# Supplementary material for: Phenotypic and Genetic Divergence among Poison Frog Populations in a Mimetic Radiation
Source: PLoS One. 2013 Feb 6;8(2):e55443. doi: 10.1371/journal.pone.0055443 (PMC3566184; doi:10.1371/journal.pone.0055443)
Supplement: Table S7 — Raw counts for relative abundance analysis. Numbers refer to the number of individuals found for that species in that site. A zero is given for cases where that species is assumed to occur at that site but was never found. Blank fields indicate the species is likely absent from that site. The superscript “m” indicates the putative model species at a given site. For the two transition zone populations, no model species is specified because the R. imitator phenotypes do not necessarily correspond to any model species. (DOCX) [file pone.0055443.s008.docx]

| **Site** | **Morph** | ***R. imitator*** | ***R. variabilis*** | ***R. summersi*** | ***R. fantastica*** |
| --- | --- | --- | --- | --- | --- |
| Curiyacu | banded | 61 | 0 | 1^m^ |  |
| Sauce | banded | 71 |  | 23^m^ |  |
| Callanayacu | transition | 16 | 1 | 0 |  |
| Chazuta | transition | 118 | 1 | 12 |  |
| Tarapoto | spotted | 28 | 1^m^ |  | 14 |
| Cainarachi valley | spotted | 124 | 72^m^ |  | 25 |
| Chumia/Shapaja | spotted | 2 | 0^m^ | 0 |  |
| Balsapuerto | striped | 3 | 0^m^ |  | 0 |
| Chipesa | striped | 59 | 1^m^ | 4 |  |
| Pongo de Cainarachi | striped | 82 | 8^m^ |  | 9 |
| Varadero | Varadero | 23 | 2 |  | 1^m^ |
